# The OASIS Consortium: integrating multi-omics technologies to transform chemical safety assessment

**DOI:** 10.1093/toxsci/kfaf128

**Published:** 2025-09-15

**Authors:** David Rouquié, Andreas Bender, Jaime Cheah, Christine E Crute, Deidre Dalmas, Jessica Ewald, Aaron Fullerton, Joshua A Harrill, Sabah Kadri, Nicole Kleinstreuer, Nynke Kramer, Jessica LaRocca, Constance A Mitchell, Srijit Seal, Shantanu Singh, Anne E Carpenter

**Affiliations:** Toxicology Data Science, Bayer SAS Crop Science Division, Sophia Antipolis, 06906, France; College of Medicine and Health Sciences, Khalifa University of Science and Technology, Abu Dhabi, P.O. Box 127788, United Arab Emirates; Centre for Molecular Informatics, Department of Chemistry, University of Cambridge, Cambridge, CB2 1EW, United Kingdom; STAR-UBB Institute, Babeş-Bolyai University, Cluj-Napoca, 400084, Romania; Center for the Development of Therapeutics, Broad Institute of MIT and Harvard, Cambridge, MA 02142, United States; Health and Environmental Sciences Institute, Washington, DC 20005, United States; Independent, Collegeville, PA 19426, United States; Imaging Platform, Broad Institute of MIT and Harvard, Cambridge, MA 02142, United States; Investigative Toxicology, Genentech, Inc, South San Francisco, CA 94080, United States; Biomolecular and Computational Toxicology Division, Center for Computational Toxicology & Exposure, Office of Research and Development, US Environmental Protection Agency, Durham, NC 27711, United States; AbbVie, North Chicago, IL 60064, United States; Division of Program Coordination, Planning, and Strategic Initiatives, National Institutes of Health, Bethesda, MD 20892, United States; Division of Toxicology, Wageningen University and Research, Wageningen, 6700 EA, The Netherlands; Corteva Agriscience, Indianapolis, IN 46268, United States; Health and Environmental Sciences Institute, Washington, DC 20005, United States; Imaging Platform, Broad Institute of MIT and Harvard, Cambridge, MA 02142, United States; Imaging Platform, Broad Institute of MIT and Harvard, Cambridge, MA 02142, United States; Imaging Platform, Broad Institute of MIT and Harvard, Cambridge, MA 02142, United States

**Keywords:** Next Generation Risk Assessment (NGRA), new approach methodologies (NAMs), Cell Painting, omics, hepatotoxicity

## Abstract

Next Generation Risk Assessment (NGRA) aims to improve safety testing of pharmaceuticals, agrochemicals, and industrial chemicals. NGRA employs new approach methodologies, such as novel in vitro assays coupled with exposure modeling, to minimize the use of animal models, which can fail to predict specific biological effects in humans. The strategy of the ‘Omics for Assessing Signatures for Integrated Safety (OASIS) Consortium combines multi-omics technologies (including transcriptomics, proteomics, and Cell Painting [high-content imaging]) and multiple cell model systems (ranging from simple cell cultures to complex organotypic models). By integrating these approaches with internal exposure estimates, the consortium aims to improve the translation between in vitro and in vivo test systems, ultimately enhancing the relevance of safety assessment to human biology. OASIS’s integrated approach aims to better translate the biological effects across different chemical and biological spaces, starting with the liver as a use case. By using compounds with well-characterized in vivo and in vitro nonclinical safety and toxicology data related to adverse organ-specific effects in rats and humans, OASIS aims to create novel integrated methods that improve safety assessment while reducing animal use. Ideally, these efforts will contribute to regulatory science across sectors and support the adoption of more predictive, efficient, and cost-effective toxicological models.

## Advancing toxicology through Next Generation Risk Assessment and multi-omics approaches: the genesis of the Omics for Assessing Signatures for Integrated Safety Consortium

Next Generation Risk Assessment (NGRA) represents a transformative shift in safety testing and toxicology. This shift spans multiple sectors, including pharmaceutical, agrochemical, and industrial chemical industries, as well as their regulatory agencies. NGRA is pushing away from traditional animal testing toward more rapid, human-relevant, and translational testing strategies using innovative new approach methodologies (NAMs) such as Cell Painting and omics technologies for a systems toxicology-based approach (Miccoli et al. 2022; Sewell et al. 2024). Animal testing, historically the gold standard, often fails to predict specific biological effects in humans accurately due to species-specific differences in target expression, pharmacological responses, toxicological responses, or disease mechanisms (Waring et al. 2015; Monticello et al. 2017; Robinson et al. 2019; Monticello et al. 2024). As a result, the field is under immense economic, ethical, societal, and scientific pressure to adopt alternative approaches to animal testing that are better aligned with 3Rs initiatives to replace, reduce, and refine the use of animals in research and that better reflect human biology and disease processes (Sewell et al. 2016).

**Fig. 1.**
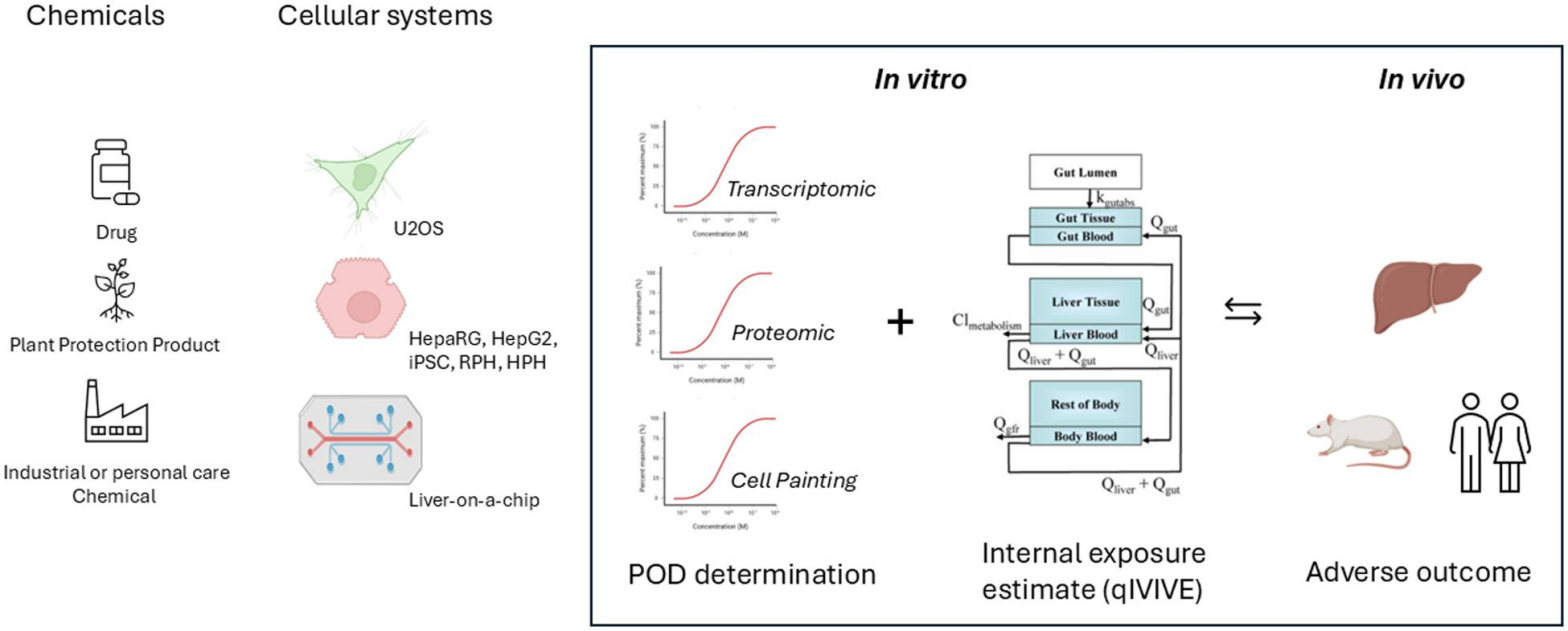
OASIS Consortium strategy. Chemical structures will be linked to different ‘omics measurements as predictive variables, as well as drug-induced liver injury (DILI) organ toxicity endpoints. The aim of the OASIS project is then to identify which in vitro proxy endpoints are predictive for in vivo toxicity, in which species, and at which dose. The aim is to establish confidence in ‘omics profiles as New Approach Methodologies and to determine best practices related to liver toxicity, with implications also for the wider field. RPH, rat primary hepatocyte; HPH, human primary hepatocyte; iPSC, induced pluripotent stem cell; qIVIVE, quantitative in vitro to in vivo extrapolation; POD, point of departure. The picture representing the internal exposure is derived from Pearce et al. (2017).

Government agencies and industry consortia are increasingly invested in NGRA and NAMs, which can play key roles in screening, prioritization, risk assessment, and mechanistic understanding of compound-induced adverse outcomes and can meet differing regulatory needs across agrochemical, pharmaceutical, and industrial sectors. For example, the US Environmental Protection Agency (EPA) and Food and Drug Administration (FDA), and the European Medicines Agency (EMA) have demonstrated commitment to NGRA and the development of NAMs via strategic plans, work groups, and industry guidelines dedicated to developing and implementing alternatives to animal testing (FDA Modernization Act 3.0 2024; USEPA 2024; USFDA 2024). The NIH Common Fund recently launched a large NAM-based biomedical research grant program to Complement Animal Research in Experimentation. Other US federal agencies, e.g. via the Interagency Coordinating Committee on the Validation of Alternative Methods (ICCVAM), are also investing substantial resources into using NAMs to improve their research portfolios and regulatory decision frameworks (NTP 2024). The European Commission is currently preparing a roadmap to phase out animal testing in chemical risk assessment (EC 2024). This situation highlights the need to enhance the translation between chemical- or drug-induced in vitro and in vivo biological responses and associated dose–response characteristics. Specifically, it is crucial to deepen our understanding of how various types of chemicals or pharmaceutical agents from different sectors interact with biological systems, from basic primary cell cultures to complex organisms, including laboratory animals and humans (Fig. 1).

To address the gap, we launched the cross-sector consortium Omics for Assessing Signatures for Integrated Safety (OASIS) to advance and evaluate the use of Cell Painting and multi-omics approaches in toxicology and risk assessment. Using liver as a test case, our objective is to explore the use and translation of biological effects from in vitro systems to laboratory animals and humans, both in terms of underlying mechanisms and concentrations or dose levels.

OASIS brings together cross-sector stakeholders to address a critical need: Developing scalable, human-relevant, and data-rich toxicological methods. This consortium aims to leverage the strengths of Cell Painting and other ‘omics technologies such as transcriptomics and proteomics. Integrating these high-dimensional data sources using machine learning strategies will hopefully bridge the gap between in vitro and in vivo models, enabling better prediction of human safety risks (Liu et al. 2023; Seal et al. 2025). OASIS seeks to enhance the prediction of liver toxicity, a key endpoint for risk assessment, as a test case, in the hopes that findings will be applicable to broader toxicological predictions.

## Evolving toxicology: from traditional animal models to high-content omics technologies using human cell-based models

Human cell-based assays offer species relevance but do not always fully replicate the complexity of in vivo human physiology (Van Norman 2019). The earliest such assays for toxicology were targeted, customized to measure a few endpoints known to be associated with particular mechanisms of toxicity. By contrast, assays that are high-dimensional, or ‘high-content’, profiling assays can produce data that capture the impact of perturbations across a broad range of molecular targets and biological processes, offering a more comprehensive and predictive approach. This strategy of measuring many transcripts, proteins, metabolites, or epigenomic modifications, each with uncertain predictive value but collectively valuable, became known as toxicogenomics (Meier et al. 2025). Initially developed to provide mechanistic insights in in vivo laboratory animal studies, toxicogenomics now supports biomarker development and dose–response assessments using in vitro test systems that aim to capture early signals of toxicity that have been identified during in vivo toxicity studies (Meier et al. 2025). Profiling technologies such as transcriptomics, Cell Painting, metabolomics, and proteomics offer high-density data and capture compound-induced changes, though they remain in early validation stages for regulatory applications such as point-of-departure determination and hazard classification (Pruteanu and Bender 2023). Additionally, both the cellular model and the technology used can affect the characterization of chemical effects (Way et al. 2022), underscoring the need to understand the biological coverage of different cell models and ‘omics platforms (Baillif et al. 2020).

OASIS chose the liver as a test case for benchmarking because it is a key organ involved in the metabolism and detoxification of chemicals, making it highly susceptible to toxicological effects (Abboud and Kaplowitz 2007). Many adverse outcomes, particularly those related to drug and chemical exposures, manifest in the liver, such as hepatotoxicity, fibrosis, and liver failure (Eaton and Klaassen 2001). Additionally, the liver’s complex metabolic activity provides critical insights into how compounds are processed and how their metabolites contribute to toxicity (Piñeiro-Carrero and Piñeiro 2004). There is also a relatively large set of publicly available in vivo data available from both rodent models and human clinical studies on liver toxicity, making it a data-rich organ for comparative analysis (Martin and Judson 2010; Igarashi et al. 2015; Chen et al. 2016; Watford et al. 2019; Daniel et al. 2022; Mezencev et al. 2024). This extensive knowledge base allows in vitro findings to be more reliably anchored to real-world outcomes, making the liver a practical and informative system for toxicological risk assessments.

### OASIS compound selection

The first task of the OASIS Consortium was to select compounds for testing across ‘omics technologies and liver cell model systems. OASIS will create a pioneering dataset by collecting and combining novel ‘omics data with existing data from standardized rat subacute in vivo toxicity studies of approximately 200 compounds generously provided by our pharmaceutical and agrochemical partners, along with in vivo toxicity data from approximately 1,500 publicly available compounds. The public OASIS compounds were selected based on publicly available subacute repeat-dose in vivo rat data or human clinical data in high-quality sources (Table 1).

**Table 1.** Sources of human data or repeated dose rat in vivo data for OASIS compounds.

Source	Website or reference	Number of compounds in OASIS list	Data type
TG-GATEs	Igarashi et al. (2015)	107	Rat in vivo
Drug Matrix	Svoboda et al. (2019)	49	Rat in vivo
ToxRefDB	Watford et al. (2019) and Feshuk et al. (2023)	102	Rat in vivo
DILIlist	FDA (2023)	875	Human Clinical
DILIRank	Chen et al. (2016)	194	Human Clinical
RepDoseDB	Bitsch et al. (2006)	241	Rat in vivo
ICE	Daniel et al. (2022)	137	Rat in vivo
Donated industry compounds	n/a	131	Rat in vivo

The consortium agreed to remove some compounds due to procurement difficulties and data availability. RepDoseDB gathered publicly available data as a database as paid service. These curated data, while in the public domain, were donated to the consortium. The compound list is available as supplemental file 1.

### OASIS cell model selection

Cell Painting, transcriptomic, and proteomic data will be collected across various liver-relevant cell systems ranging from simple cultured cell lines to primary hepatocytes and advanced models such as liver organoids and liver-on-a-chip platforms (Table 2). Cell Painting will be conducted across all compounds and cell lines; however, due to cost, the transcriptomic and proteomic data generation will be limited to a subset of compounds, as will the advanced cell models. The specific advanced cell models remain to be selected by OASIS.

**Table 2.** OASIS cell models.

Cell model	Description	Metabolic capability	Rationale
U2OS	Human osteosarcoma immortal cell line	Very limited metabolic capability	Used to detect basic cytotoxicity and identify pathway activity using public JUMP-Cell Painting Consortium data
HepG2*	Human hepatocellular carcinoma cell line	Limited metabolic capability	Commonly used for cytotoxicity and drug metabolism studies
HepaRG	Human primary hepatocyte-like cell line	Many intact metabolic pathways	Selected as a cost-effective human-derived cell line with stable liver-like functions, offering a practical alternative to primary hepatocytes for routine testing
Rat primary hepatocytes*	Rat primary hepatocyte cells	Intact metabolic pathways	Included for their species-specific metabolic capabilities to benchmark in vitro data against established rat in vivo toxicity outcomes
Huiman primary hepatocytes*	Human primary hepatocyte cells	Intact metabolic pathways	Selected as the gold standard for human liver metabolism and toxicity studies due to their intact metabolic and functional pathways
iPSC-derived human liver organoids*	Hepatocytes, stellate cells, and Kupffer cells	Partial liver functionality	Provides a multicellular, physiologically relevant model for liver toxicity
Liver-on-a-chip*	Rat and human liver cells	Species-specific metabolic pathways	Offers an advanced, species-specific system for studying dynamic liver functions and toxicity under more physiologically relevant conditions

The selected cell models are relevant to humans or rats, ranging in complexity from well-established cell lines to more complex primary hepatocytes, to organoids and liver-on-a-chip devices.

*Due to resource constraints, varying subsets of the full compound set will be tested in these cell models.

### OASIS ‘omics technologies

In this and subsequent sections, we evaluate the status of key profiling methods in rat and human in vitro cellular systems, with a focus on the utility of technologies selected for the OASIS Consortium and their ability to translate to in vivo rat and human known adverse outcomes.

### Transcriptomics

Transcriptomics measures mRNA levels in cell cultures, organs, and biological fluids; in toxicology, these data are compared with controls to characterize and quantify changes in gene expression in response to a chemical. Techniques range from targeted measurement of a small number of transcripts (RT-qPCR) to broader profiling approaches such as microarrays and Next Generation RNA Sequencing. However, linking gene expression changes—indicative of compound toxicity or bioactivity—with observable outcomes (e.g. liver injury) is complex. Standard bioinformatics outputs, such as lists of differentially expressed genes, are challenging to integrate into safety assessment frameworks because they must be related to dose responses and correlated with pathological responses in humans. Regarding industrial chemicals, 1 quantitative emerging approach is to summarize ‘omics concentration–response data as a point of departure (POD—the lowest concentration at which a compound causes a statistically significant change in the mRNA profile) based on transcriptomic profiling, producing a benchmark dose from animal studies that naturally fit within a human health assessment framework (Auerbach et al. 2024). High-throughput transcriptomics using cell line cultures has also been used to determine potencies associated with perturbation of biological pathways that may underlie (Harrill et al. 2019; Yauk et al. 2020; Meier et al. 2025). However, this approach is not widely used across other sectors, such as the pharmaceutical or agrochemical industry, for regulatory decision-making due to limitations related to standardization, data interpretation, and the complexity of integrating transcriptomic data with existing regulatory frameworks (Harrill et al. 2021; Gant et al. 2023). That said, pathway-specific transcriptomics signatures (e.g. TGX-DDI, GARD skin) have demonstrated successful applications (Johansson et al. 2019; Li et al. 2019), but widespread adoption across industries and for regulatory decision-making remain limited in part due to the lack of a broad validation effort.

### Proteomics

Proteomics data promises to provide a more direct understanding of cellular perturbations caused by chemicals than mRNA for 2 key reasons. First, proteins frequently carry out the functions encoded by genes (Liang et al. 2020). Second, molecular initiating events and key events (2 categories of toxicity mechanisms) are more often defined with respect to proteins than to the mRNAs that encode them. However, mass spectrometry or ELISA-based proteomics methods have historically been too expensive and low-throughput for analysis beyond a few compounds, which is insufficient for benchmarking broader predictive ability. Proteomics recently revealed the dose- and time-resolved pharmacological properties of a limited number of drugs (Zecha et al. 2023), but new methods have recently become cost-effective enough to analyze sufficient numbers of compounds, in concentration–response, to quantitatively evaluate whether the technologies can predict toxicity. Because proteomics data can be analyzed in the same way as transcriptomics data, the OASIS Consortium provides a unique opportunity to evaluate proteomics data and integration with Cell Painting and transcriptomics data to better predict the potential for toxicity, point-of-departure identification, and biomarker identification and interpretation.

### Cell painting

Cell Painting is an in vitro phenotypic profiling assay that labels cells with 6 fluorescent probes to visualize 8 different cellular components by microscopy (Seal et al. 2024). Image analysis software measures thousands of features, including intensity, shape, textures, and so on, from each cell. Cell Painting is cost-effective and high-throughput, yet it captures data at single-cell resolution, allowing for assessment of population heterogeneity and providing information that is distinct from mRNA and protein profiling (Wawer et al. 2014; Way et al. 2022; Dahlin et al. 2023; Dagher et al. 2025). Cell Painting can discern distinct mechanisms of chemical cytotoxicity (Way et al. 2022; Dahlin et al. 2023), determine potencies for perturbation of cellular biology (Nyffeler et al. 2020; Nyffeler et al. 2021; Way et al. 2022; Ewald et al. 2025), and group chemicals acting through similar mechanism (Nyffeler et al. 2023). Phenotypic profiling approaches like Cell Painting have been explored to predict lung and kidney toxicity (Su et al. 2016; Lee et al. 2018), and efforts to better understand the applicability of Cell Painting for toxicity assessment across sectors are still underway. The OASIS Consortium focuses on the applicability and incorporation of this technology for application to safety risk assessment, which has not yet been fully explored (Chavan et al. 2020; Lejal et al. 2023).

## Quantitative in vitro to in vivo extrapolation: bridging laboratory and real-world exposures

A critical challenge in toxicology is translating laboratory findings to real-world relevance. Quantitative in vitro to in vivo extrapolation (qIVIVE) addresses this by converting the concentrations that cause effects in laboratory cell cultures to the exposure levels that would produce equivalent effects in living organisms.

This translation requires 2 key components: First, accurate measurement of the biologically active concentration in cell-based assays, and second, pharmacokinetic modeling to predict what dose would achieve those tissue concentrations in humans or animals. OASIS will integrate qIVIVE modeling with multi-omics data to enhance the prediction of human liver toxicity from laboratory studies, providing a quantitative bridge between in vitro observations and in vivo outcomes (Chang et al. 2022; Magurany et al. 2023; Moreau et al. 2024).

## OASIS Consortium

‘Omics technologies and in vitro models coupled with exposure modeling offer promise but need to be evaluated to gain a deeper understanding of their capacities and limitations for human health risk assessment. The scale of the challenge requires pre-competitive collaboration among industries, regulators, and academia. Creating a sufficiently scaled validation dataset to evaluate ‘omics technologies, sharing insights on translating in vitro to in vivo models, integrating multi-omics data, and deriving mechanistic understanding will build trust and advance the adoption of NAMs in chemical risk assessment. To that end, participating organizations in the OASIS Consortium span sectors (pharmaceuticals, agrochemicals, public research, and governmental organizations) and disciplines (toxicology, ‘omics, drug discovery, bioinformatics, in vitro technologies, and Physiologically Based Pharmacokinetic modeling). An up-to-date list of participating organizations can be found at https://oasisconsortium.org/members/.

OASIS aims to overcome 3 major existing limitations to creating better, faster, and less costly methods for toxicity assessment: (i) the lack of reasonably scalable assay systems that retain physiological relevance to organ systems while also producing multiplexed, high-dimensional readouts, which we overcome here via several newer profiling methods (Cell Painting, targeted transcriptomics, and multiplexed affinity proteomics), (ii) the small number of compounds with publicly available in vivo toxicity outcome data, which we increase here through (a) a concerted effort to gather such a set of compounds with public toxicity data, plus (b) the industry partners’ contributions of compounds with existing, previously private data, and (iii) the challenge of translating in vitro dose–response data to organisms, which we address with experts in pharmacokinetics.

OASIS key guiding questions are as follows:

- How do different ‘omics technologies capture, from in vitro cell cultures, various modes of action in informing adverse outcomes in rats and humans, and how do these outcomes relate to the chemical spaces of agricultural chemicals, industrial chemicals, and pharmaceuticals, given their distinct characteristics? Does Cell Painting add value to other ‘omics-based assessments enabling better in vitro to in vivo translation?
- How do the physiological relevancies of cellular models—such as cell lines, primary cell cultures, and organ-on-chip systems—compare in predicting human outcomes, and how can further comparisons with rat models provide insight into species-specific differences?
- To what extent do in vitro biological responses, combined with internal exposure estimates (e.g. qIVIVE), inform the dose–response relationship for in vivo adverse outcomes, and how do PODs derived from different ‘omics technologies and Cell Painting compare in this context?

## Benchmarking ‘omics data using liver toxicity models: cross-species comparisons and predictive assessments

OASIS is comprehensively benchmarking a diverse set of 1513 data-rich compounds, including 963 compounds previously tested in many studies as part of ToxCast (Richard et al. 2016). These compounds serve as a critical foundation for understanding adverse outcomes, drawing from both publicly available sources and contributions from industry partners. Many of the compounds are labeled with categorical classifications of adverse effects, such as the presence or absence of drug-induced liver injury (DILI) in humans, rather than detailed dose–response profiles across species. For compounds with data from both rat models and human studies, the aim is to evaluate cross-species concordance through both qualitative and quantitative analyses.

**Fig. 2.**
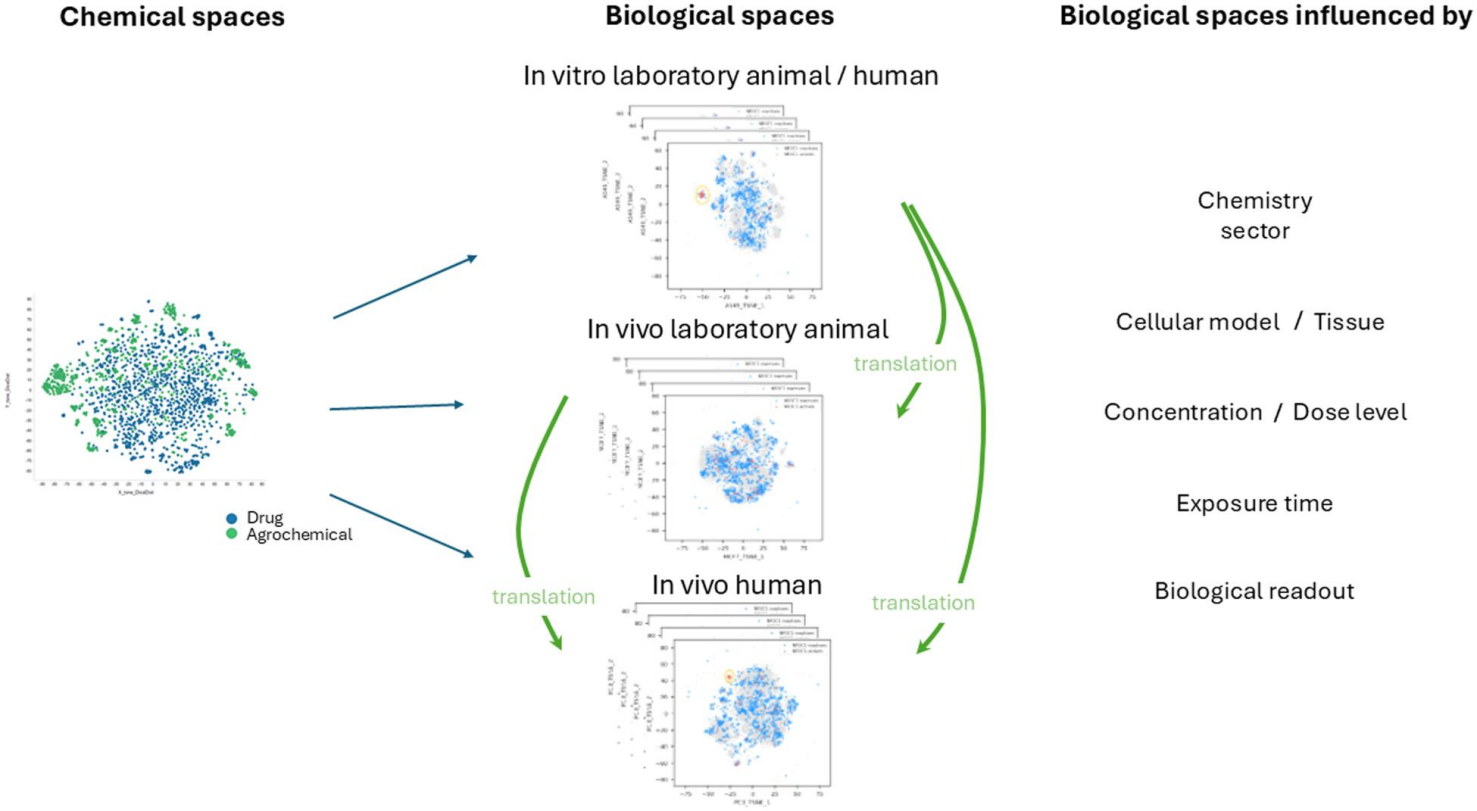
Hypothetical representations of chemical and biological spaces illustrate how chemical compounds can elicit varying responses across different experimental systems—such as in vitro models, in vivo animal studies, and human biology. These biological effects are influenced by multiple factors, including the chemical structure, the species or cell model used, the duration and concentration of exposure, and the specific biological endpoint being measured. Understanding how responses translate across these spaces is crucial for accurately predicting human outcomes and improving the relevance of preclinical models.

Our benchmarking strategy incorporates rigorous, quantifiable metrics to assess the translation of in vitro findings to in vivo outcomes (Fig. 2). Specifically, we will evaluate the concordance between in vitro PODs for each combination of omics and cell model tested, coupled with in silico exposure estimates, and their known in vivo counterparts, using robust statistical methods, establishing thresholds to determine predictive reliability. These quantitative benchmarks are essential for selecting and building confidence in the predictive power of in vitro models and readouts, as well as understanding their limitations, ultimately enhancing their utility in risk assessment and regulatory decision-making. These benchmarks will also help prioritize future investments to fill gaps in performance. It should be noted that while we will compare human cell-derived PODs and rat cell-derived PODs, we will also assess the mechanistic insights derived from human and rat in vitro test systems. In many instances, there are notable species differences in response to outcomes, along with expected ADME differences between in vivo and in vitro. Although these PODs will help us benchmark outcomes, there will be nuance in understanding and interpreting results.

## Future directions: enhancing translational toxicology

In summary, OASIS will investigate the effectiveness of several ‘omic profiles measured from several in vitro cell models in predicting organ-level responses in both rats and humans. We will evaluate which ‘omics technologies are most indicative of which organ-specific effects, with the goal of refining toxicity testing strategies. A crucial outcome will be the improved ability to extrapolate data between species, thereby enhancing the translational accuracy of these models. Furthermore, OASIS aims to identify the ‘omics readouts that, when integrated with each other and with qIVIVE, reliably predict clinical DILI risk early in the development of pharmaceutical, agrochemical, and industrial products. All data and results will be made publicly accessible upon publication of relevant papers to promote transparency and collaboration.

This initial phase of work is focusing on the information obtained across different ‘omic methods and cell types related to liver. Looking forward, the integration of additional cell types into toxicology studies will broaden the applicability of these approaches, extending insights beyond liver toxicity. Validation efforts, such as ring trials and the standardization of methodologies across laboratories, will be critical for ensuring reproducibility and gaining regulatory acceptance. Incorporating other ‘omics technologies, like metabolomics, will have the potential to further enhance mechanistic understanding, and expanding research into non-liver toxicities and ecotoxicological endpoints will diversify the risk assessment landscape. Later phases of work may also include understanding how these tools and other NAMs can be used for hazard identification or risk assessment.

Ultimately, this research aims to bridge the gap between preclinical findings and clinical outcomes, reducing the likelihood of late-stage failures in drug development and protecting human health in other industries. By providing robust, at scale evidence-based data to better understand the translation between in vitro and in vivo test systems, ultimately enhancing the relevance of safety assessment to human biology, OASIS will advance NGRA by characterizing methods that could provide alternatives to animal testing while maintaining rigorous safety standards.

## Supplementary Material

kfaf128_Supplementary_Data

## References

[kfaf128-B1] Abboud G , Kaplowitz N. 2007. Drug-induced liver injury. Drug Saf. 30:277–294. 10.2165/00002018-200730040-00001. 17408305

[kfaf128-B2] Auerbach S , Casey W, Chang D, Cowden J, Davidson-Fritz S, Dean J, DeVito M, Everett L, Harrill AH, Hester S, et al 2024. US Environmental Protection Agency Center for Computational Toxicology and Exposure’s Reports. In: Standard methods for development of EPA transcriptomic assessment products (ETAPs). U.S. Environmental Protection Agency. 10.23645/epacomptox.25365496. 39186581

[kfaf128-B3] Baillif B , Wichard J, Méndez-Lucio O, Rouquié D. 2020. Exploring the use of compound-induced transcriptomic data generated from cell lines to predict compound activity toward molecular targets [original research]. Front Chem. 8:296. 10.3389/fchem.2020.00296. 32391323 PMC7191531

[kfaf128-B4] Chang X , Tan YM, Allen DG, Bell S, Brown PC, Browning L, Ceger P, Gearhart J, Hakkinen PJ, Kabadi SV, et al 2022. IVIVE: facilitating the use of in vitro toxicity data in risk assessment and decision making. Toxics. 10:232. 10.3390/toxics10050232. PMC9143724 35622645

[kfaf128-B5] Chavan S , Scherbak N, Engwall M, Repsilber D. 2020. Predicting chemical-induced liver toxicity using high-content imaging phenotypes and chemical descriptors: a random forest approach. Chem Res Toxicol. 33:2261–2275. 10.1021/acs.chemrestox.9b00459. 32830476

[kfaf128-B6] Chen M , Suzuki A, Thakkar S, Yu K, Hu C, Tong W. 2016. DILIrank: the largest reference drug list ranked by the risk for developing drug-induced liver injury in humans. Drug Discov Today. 21:648–653. 10.1016/j.drudis.2016.02.015. 26948801

[kfaf128-B42921880] Dagher M, Ongo G, Robichaud N, Kong J, Rho W, Teahulos I, Tavakoli A, Bovaird S, Merjaneh S, Tan A, et al 2025. nELISA: A high-throughput, high-plex platform enables quantitative profiling of the inflammatory secretome. bioRxiv. 2025;2023.04.17.535914. 10.1101/2023.04.17.535914, preprint: not peer reviewed. PMC12615254 41203862

[kfaf128-B4292188] Dahlin JL, , Hua BK, , Zucconi BE, , Nelson SD, , Singh S, , Carpenter AE, , Shrimp JH, , Lima-Fernandes E, , Wawer MJ, , Chung LPW, et al 2023. Reference compounds for characterizing cellular injury in high-content cellular morphology assays. Nat Commun. 14:1364. 10.1038/s41467-023-36829-x PMC10011410 36914634

[kfaf128-B7] Daniel AB , Choksi N, Abedini J, Bell S, Ceger P, Cook B, Karmaus AL, Rooney J, To KT, Allen D, et al 2022. Data curation to support toxicity assessments using the Integrated Chemical Environment. Front Toxicol. 4:987848. 10.3389/ftox.2022.987848. 36408349 PMC9669273

[kfaf128-B8] Eaton DL , Klaassen CD. 2001. Principles of toxicology. In: Klaassen CD, editor. Casarett and Doull’s toxicology: the basic science of poisons. Vol. 6. New York: McGraw-Hill.

[kfaf128-B9] EC. 2024. Animal testing in chemical safety assessments: commission roadmap to phase it out. [accessed 2005 Jun 1]. https://ec.europa.eu/info/law/better-regulation/have-your-say/initiatives/14281-Animal-testing-in-chemical-safety-assessments-Commission-roadmap-to-phase-it-out_en.

[kfaf128-B10] Ewald JD , Titterton KL, Bauerle A, Beatson A, Boiko DA, Cabrera AA, Cheah J, Cimini BA, Gorissen B, Jones T, et al 2025. Cell painting for cytotoxicity and mode-of-action analysis in primary human hepatocytes. bioRxiv. 10.1101/2025.01.22.634152, preprint: not peer reviewed. PMC13725082 41903531

[kfaf128-B11] FDA Modernization Act 3.0. 2024. [accessed 2005 Aug 1]. https://www.congress.gov/bill/118th-congress/house-bill/7248/text.

[kfaf128-B12] Gant TW , Auerbach SS, Von Bergen M, Bouhifd M, Botham PA, Caiment F, Currie RA, Harrill J, Johnson K, Li D, et al 2023. Applying genomics in regulatory toxicology: a report of the ECETOC workshop on omics threshold on non-adversity. Arch Toxicol. 97:2291–2302. 10.1007/s00204-023-03522-3. 37296313 PMC10322787

[kfaf128-B13] Harrill J , Shah I, Setzer RW, Haggard D, Auerbach S, Judson R, Thomas RS. 2019. Considerations for strategic use of high-throughput transcriptomics chemical screening data in regulatory decisions. Curr Opin Toxicol. 15:64–75. 10.1016/j.cotox.2019.05.004. 31501805 PMC6733036

[kfaf128-B14] Harrill JA , Viant MR, Yauk CL, Sachana M, Gant TW, Auerbach SS, Beger RD, Bouhifd M, O'Brien J, Burgoon L, et al 2021. Progress towards an OECD reporting framework for transcriptomics and metabolomics in regulatory toxicology. Regul Toxicol Pharmacol. 125:105020. 10.1016/j.yrtph.2021.105020. 34333066 PMC8808338

[kfaf128-B15] Igarashi Y , Nakatsu N, Yamashita T, Ono A, Ohno Y, Urushidani T, Yamada H. 2015. Open TG-GATEs: a large-scale toxicogenomics database. Nucleic Acids Res. 43:D921–D927. 10.1093/nar/gku955. 25313160 PMC4384023

[kfaf128-B9739061] Johansson H, , Gradin R, , Johansson A, , Adriaens Els, , Edwards A, , Zuckerstätter V, , Jerre A, , Burleson F, , Gehrke H, , Roggen EL. 2019. Validation of the gard™skin assay for assessment of chemical skin sensitizers: Ring trial results of predictive performance and reproducibility. Toxicol Sci. 170: 374–381. 10.1093/toxsci/kfz108 31099396 PMC6657565

[kfaf128-B15619699] Lee J, , Miller JA, , Basu S, , Kee V, , Loo L-H. 2018. Building predictive in vitro pulmonary toxicity assays using high-throughput imaging and artificial intelligence. Arch Toxicol. 92: 2055–2075. 10.1007/s00204-018-2213-0 29705884 PMC6002469

[kfaf128-B16] Lejal V , Cerisier N, Rouquié D, Taboureau O. 2023. Assessment of drug-induced liver injury through cell morphology and gene expression analysis. Chem Res Toxicol. 36:1456–1470. 10.1021/acs.chemrestox.2c00381. 37652439 PMC10523580

[kfaf128-B8266443] Li H-H, , Yauk CL, , Chen R, , Hyduke DR, , Williams A, , Frötschl R, , Ellinger-Ziegelbauer H, , Pettit S, , Aubrecht J, , Fornace AJ. 2019. TGx-DDI, a transcriptomic biomarker for genotoxicity hazard assessment of pharmaceuticals and environmental chemicals. Front Big Data. 2:36. 10.3389/fdata.2019.00036 PMC7931968 33693359

[kfaf128-B17] Liang X , Martyniuk CJ, Simmons DBD. 2020. Are we forgetting the “proteomics” in multi-omics ecotoxicology? Comp Biochem Physiol Part D Genomics Proteomics. 36:100751. 10.1016/j.cbd.2020.100751. 33142247

[kfaf128-B18] Liu A , Seal S, Yang H, Bender A. 2023. Using chemical and biological data to predict drug toxicity. SLAS Discov. 28:53–64. 10.1016/j.slasd.2022.12.003. 36639032

[kfaf128-B19] Magurany KA , Chang X, Clewell R, Coecke S, Haugabrooks E, Marty S. 2023. A pragmatic framework for the application of new approach methodologies in one health toxicological risk assessment. Toxicol Sci. 192:155–177. 10.1093/toxsci/kfad012. 36782355 PMC10109535

[kfaf128-B20] Martin MT , Judson R. 2010. ToxRefDB—release user-friendly web-based tool for mining ToxRefDB. U.S. Environmental Protection Agency.

[kfaf128-B21] Meier MJ , Harrill J, Johnson K, Thomas RS, Tong W, Rager JE, Yauk CL. 2025. Progress in toxicogenomics to protect human health. Nat Rev Genet. 26:105–122. 10.1038/s41576-024-00767-1. 39223311

[kfaf128-B22] Mezencev R , Feshuk M, Kolaczkowski L, Peterson GC, Zhao QJ, Watford S, Weaver JA. 2024. The association between histopathologic effects and liver weight changes induced in mice and rats by chemical exposures: an analysis of the data from Toxicity Reference Database (ToxRefDB). Toxicol Sci. 200:404–413. 10.1093/toxsci/kfae056. 38656946 PMC12021267

[kfaf128-B23] Miccoli A , Marx-Stoelting P, Braeuning A; German Federal Institute for Risk Assessment (BfR), Berlin, Germany. 2022. The use of NAMs and omics data in risk assessment. EFSA J. 20:e200908. 10.2903/j.efsa.2022.e200908. 36531284 PMC9749445

[kfaf128-B24] Monticello TM , Jones TW, Dambach DM, Potter DM, Bolt MW, Liu M, Keller DA, Hart TK, Kadambi VJ. 2017. Current nonclinical testing paradigm enables safe entry to First-In-Human clinical trials: the IQ consortium nonclinical to clinical translational database. Toxicol Appl Pharmacol. 334:100–109. 10.1016/j.taap.2017.09.006. 28893587

[kfaf128-B25] Monticello TM , Potter DM, Huang Q, Hart TK, Shuey D, Troth S, Vergis JM, Tassew N, Glascott P. 2024. Do longer duration nonclinical toxicology studies provide predictive clinical safety value? The IQ consortium longer duration nonclinical to clinical translational database. Toxicol Appl Pharmacol. 492:117087. 10.1016/j.taap.2024.117087. 39243825

[kfaf128-B26] Moreau M , Simms L, Andersen ME, Trelles Sticken E, Wieczorek R, Pour SJ, Chapman F, Roewer K, Otte S, Fisher J, et al 2024. Use of quantitative in vitro to in vivo extrapolation (QIVIVE) for the assessment of non-combustible next-generation product aerosols [original research]. Front Toxicol. 6:1373325. 10.3389/ftox.2024.1373325. 38665213 PMC11043521

[kfaf128-B27] NTP. 2024. ICCVAM—Interagency Coordinating Committee on the Validation of Alternative Methods. [accessed 2005 Aug 1]. https://ntp.niehs.nih.gov/go/iccvam.

[kfaf128-B28] Nyffeler J , Haggard DE, Willis C, Setzer RW, Judson R, Paul-Friedman K, Everett LJ, Harrill JA. 2021. Comparison of approaches for determining bioactivity hits from high-dimensional profiling data. SLAS Discov. 26:292–308. 10.1177/2472555220950245. 32862757 PMC8673120

[kfaf128-B29] Nyffeler J , Willis C, Harris FR, Foster MJ, Chambers B, Culbreth M, Brockway RE, Davidson-Fritz S, Dawson D, Shah I, et al 2023. Application of cell painting for chemical hazard evaluation in support of screening-level chemical assessments. Toxicol Appl Pharmacol. 468:116513. 10.1016/j.taap.2023.116513. 37044265 PMC11917499

[kfaf128-B30] Nyffeler J , Willis C, Lougee R, Richard A, Paul-Friedman K, Harrill JA. 2020. Bioactivity screening of environmental chemicals using imaging-based high-throughput phenotypic profiling. Toxicol Appl Pharmacol. 389:114876. 10.1016/j.taap.2019.114876. 31899216 PMC8409064

[kfaf128-B9539229] Pearce RG, , Setzer RW, , Strope CL, , Sipes NS, , Wambaugh JF. 2017. httk : R package for high-throughput toxicokinetics. J Stat Soft. 79:1-26. 10.18637/jss.v079.i04 PMC6134854 30220889

[kfaf128-B31] Piñeiro-Carrero VM , Piñeiro EO. 2004. Liver. Pediatrics. 113:1097–1106. 15060205

[kfaf128-B32] Pruteanu L-L , Bender A. 2023. Using transcriptomics and cell morphology data in drug discovery: the long road to practice. ACS Med Chem Lett. 14:386–395. 10.1021/acsmedchemlett.3c00015. 37077392 PMC10107910

[kfaf128-B33] Richard AM , Judson RS, Houck KA, Grulke CM, Volarath P, Thillainadarajah I, Yang C, Rathman J, Martin MT, Wambaugh JF, et al 2016. ToxCast chemical landscape: paving the road to 21st century toxicology. Chem Res Toxicol. 29:1225–1251. 10.1021/acs.chemrestox.6b00135. 27367298

[kfaf128-B34] Robinson NB , Krieger K, Khan FM, Huffman W, Chang M, Naik A, Yongle R, Hameed I, Krieger K, Girardi LN, et al 2019. The current state of animal models in research: a review. Int J Surg. 72:9–13. 10.1016/j.ijsu.2019.10.015. 31627013

[kfaf128-B35] Seal S , Mahale M, Garcia-Ortegon M, Joshi CK, Hosseini-Gerami L, Beatson A, Greenig M, Shekhar M, Patra A, Weis C, et al 2025. Machine learning for toxicity prediction using chemical structures: pillars for success in the real world. Chem Res Toxicol. 38:759–807. 10.1021/acs.chemrestox.5c00033. 40314361 PMC12093382

[kfaf128-B36] Seal S , Trapotsi MA, Spjuth O, Singh S, Carreras-Puigvert J, Greene N, Bender A, Carpenter AE. 2024. A decade in a systematic review: the evolution and impact of cell painting. bioRxiv. 10.1101/2024.05.04.592531, preprint: not peer reviewed.

[kfaf128-B37] Sewell F , Alexander-White C, Brescia S, Currie RA, Roberts R, Roper C, Vickers C, Westmoreland C, Kimber I. 2024. New approach methodologies (NAMs): identifying and overcoming hurdles to accelerated adoption. Toxicol Res (Camb). 13:tfae044. 10.1093/toxres/tfae044. 38533179 PMC10964841

[kfaf128-B38] Sewell F , Edwards J, Prior H, Robinson S. 2016. Opportunities to apply the 3Rs in safety assessment programs. ILAR J. 57:234–245. 10.1093/ilar/ilw024. 28053076 PMC5886346

[kfaf128-B61681272] Su R, , Xiong S, , Zink D, , Loo L-H. 2016. High-throughput imaging-based nephrotoxicity prediction for xenobiotics with diverse chemical structures. Arch Toxicol. 90:2793–2808. 10.1007/s00204-015-1638-y 26612367 PMC5065616

[kfaf128-B39] USEPA. 2024. EPA new approach methods work plan: reducing the use of vertebrate animals in chemical testing. [accessed 2005 Aug 1]. https://www.epa.gov/chemical-research/epa-new-approach-methods-work-plan-reducing-use-vertebrate-animals-chemical.

[kfaf128-B40] USFDA. 2024. Advancing alternative methods at FDA. [accessed 2005 Aug 1]. https://www.fda.gov/science-research/about-science-research-fda/advancing-alternative-methods-fda.

[kfaf128-B41] Van Norman GA. 2019. Limitations of animal studies for predicting toxicity in clinical trials: is it time to rethink our current approach? JACC Basic Transl Sci. 4:845–854. 10.1016/j.jacbts.2019.10.008. 31998852 PMC6978558

[kfaf128-B42] Waring MJ , Arrowsmith J, Leach AR, Leeson PD, Mandrell S, Owen RM, Pairaudeau G, Pennie WD, Pickett SD, Wang J, et al 2015. An analysis of the attrition of drug candidates from four major pharmaceutical companies. Nat Rev Drug Discov. 14:475–486. 10.1038/nrd4609. 26091267

[kfaf128-B43] Watford S , Ly Pham L, Wignall J, Shin R, Martin MT, Friedman KP. 2019. ToxRefDB version 2.0: improved utility for predictive and retrospective toxicology analyses. Reprod Toxicol. 89:145–158. 10.1016/j.reprotox.2019.07.012. 31340180 PMC6944327

[kfaf128-B4154636] Wawer MJ, , Jaramillo DE, , Dančík V, , Fass DM, , Haggarty SJ, , Shamji AF, , Wagner BK, , Schreiber SL, , Clemons PA. 2014. Automated structure–activity relationship mining: Connecting chemical structure to biological profiles. SLAS Discovery. 19: 738–748. 10.1177/1087057114530783 PMC5554950 24710340

[kfaf128-B48395920] Way GP, , Natoli Ted, , Adeboye A, , Litichevskiy Lev, , Yang A, , Lu X, , Caicedo JC, , Cimini BA, , Karhohs K, , Logan DJ, et al 2022. Morphology and gene expression profiling provide complementary information for mapping cell state. Cell Syst. 13: 911–923.e9. 10.1016/j.cels.2022.10.001 36395727 PMC10246468

[kfaf128-B45] Yauk CL , Harrill AH, Ellinger-Ziegelbauer H, van der Laan JW, Moggs J, Froetschl R, Sistare F, Pettit S. 2020. A cross-sector call to improve carcinogenicity risk assessment through use of genomic methodologies. Regul Toxicol Pharmacol. 110:104526. 10.1016/j.yrtph.2019.104526. 31726190 PMC7891877

[kfaf128-B46] Zecha J , Bayer FP, Wiechmann S, Woortman J, Berner N, Müller J, Schneider A, Kramer K, Abril-Gil M, Hopf T, et al 2023. Decrypting drug actions and protein modifications by dose- and time-resolved proteomics. Science. 380:93–101. 10.1126/science.ade3925. 36926954 PMC7615311

